# Core Patient-Centered Outcomes for Adolescents and Young Adults with Cancer: A Comprehensive Review of the Literature from the STRONG-AYA Project

**DOI:** 10.3390/cancers17030454

**Published:** 2025-01-28

**Authors:** Silvie H. M. Janssen, Winette T. A. van der Graaf, Anna Hurley-Wallace, Carla Vlooswijk, Catarina S. Padilla, Charlotte Cairns, Connor J. Tyler, Emily I. Holthuis, Gudrun E. Rohde, Katherine J. Hunt, Kirsty Way, Konstantinos Angoumis, Lina H. Lankhorst, Maarten F. M. Engel, Maria-Sophie Rothmund, Milou J. P. Reuvers, Nicole Collaço, Richard Wagland, Samantha C. Sodergren, Simone Hanebaum, Thom Legendal, Thomas J. Cartledge, Tom I. Bootsma, Yushi Bai, Anne-Sophie Darlington, Olga Husson

**Affiliations:** 1 Department of Medical Oncology, Netherlands Cancer Institute, Plesmanlaan 121, 1066 CX Amsterdam, The Netherlands; sh.janssen@nki.nl (S.H.M.J.);; 2 Department of Medical Oncology, Erasmus MC Cancer Institute, Erasmus University Medical Center, 3015 GD Rotterdam, The Netherlands; 3 Department of Public Health, Erasmus MC Cancer Institute, Erasmus University Medical Center, Doctor Molewaterplein 40, 3015 GD Rotterdam, The Netherlands; 4 School of Health Sciences, University of Southampton, Southampton SO17 1BJ, UKc.tyler@soton.ac.uk (C.J.T.);; 5 Research and Development, Netherlands Comprehensive Cancer Organization, 3511 CV Utrecht, The Netherlands; 6 Department of Health and Nursing Science, Faculty of Health and Sport Sciences, University in Agder, 4604 Kristiansand, Norway; 7 Marie Curie Palliative Care Research Department, Division of Psychiatry, University College London, London W1T 7NF, UK; 8 Department of Clinical Research, Sorlandet Hospital, 4615 Kristiansand, Norway; 9 Medical Library, Erasmus MC, Erasmus University Medical Center, 3015 GD Rotterdam, The Netherlands; 10 Department of Psychiatry, Psychotherapy, Psychosomatics, and Medical Psychology, University Hospital of Psychiatry II, Medical University of Innsbruck, 6020 Innsbruck, Austria; 11 Faculty of Medicine, Academic Geriatric Medicine, University of Southampton, Southampton SO17 1BJ, UK; 12 Division of Psychology and Mental Health, Faculty of Biology, Medicine and Health, University of Manchester, Manchester M13 9PL, UK; 13 Department of Surgical Oncology, Erasmus MC Cancer Institute, Erasmus University Medical Center, Doctor Molewaterplein 40, 3015 GD Rotterdam, The Netherlands

**Keywords:** adolescents and young adults, AYAs, cancer, core outcome set, literature review

## Abstract

Background: In adolescents and young adults (AYAs) with cancer, the importance of patient-centered research outcomes is increasingly acknowledged and there is a need for consensus on the most relevant outcomes. This review provides a comprehensive overview of outcomes relevant to AYAs with cancer in order to develop an AYA-specific core outcome set (COS) as part of the European project STRONG-AYA. Methods: A literature search was conducted to identify relevant articles in five databases. Each article was reviewed by two independent researchers, using Rayyan (an online tool) to screen articles by their title/abstract and then by their full text. Subsequently, the data were extracted using a predefined, tailored extraction framework. Results: In total, 17,301 articles were screened, of which 1631 were included in the analyses. Registry studies (52.9%) were the most used study type. Well-represented continents were North America (50.3%) and Europe (27.8%). Of the five core areas, functioning (47.0%) and epidemiology (44.9%) were covered most often, followed by physiological/clinical (42.4%), resource use (6.1%), and adverse events (4.5%). The outcome domains that were represented the most include mortality/survival (43.1%), delivery of care (23.4%), outcomes relating to neoplasms (22.7%), and emotional functioning/well-being (19.2%). Conclusions: This literature review provides a foundation for the development of an AYA-specific COS for improving the relevance and efficiency of measuring outcomes, the pooling of (international) research data, and the value of care for current and future AYAs with cancer. Finalization and refinement of the COS should be a priority for future research.

## Simple Summary

This review provides an overview of relevant outcomes of AYAs with cancer in order to develop a core outcome set (COS: an agreed standardized collection of outcomes) for this population. A literature search was conducted, after which articles were reviewed by two independent researchers using Rayyan to screen articles by their title/abstract and their full text. The data were extracted using a tailored extraction framework by a team of reviewers. A total of 1631 of the 17,301 screened articles were included. Of the five core areas, functioning (47.0%) and epidemiology (44.9%) were covered most often, followed by physiological/clinical (42.4%), resource use (6.1%), and adverse events (4.5%). The most represented outcome domains include mortality/survival, delivery of care, outcomes relating to neoplasms, and emotional functioning/well-being. This literature review provides a foundation for the development of an AYA-specific COS to improve the relevance and efficiency of measuring outcomes, the pooling of (international) data, and the value of care for AYAs with cancer.

## 1. Introduction

Adolescents and young adults (AYAs) with cancer, internationally defined as aged 15–39 years old at initial cancer diagnosis, form a distinct population within oncology [1]. They differ from pediatric (<15 years) and older adult (>39 years) cancer patients based on tumor epidemiology and biology, treatment, needs, and their developmental life phase [1,2,3,4,5,6]. The latter refers to unique milestones that are representative of this age period, including graduating from school, building a career, forming romantic relationships and a family, and becoming (financially) independent. A cancer diagnosis and subsequent treatment at this age may delay or even prevent these milestones from being achieved. In addition, AYAs with cancer are at risk of long-term and late effects, which may also affect the quality and quantity of their survival [7,8,9].

Due to the (global) burden of cancer among AYAs, there is a growing group of AYAs whose needs are unmet as they are poorly served by either pediatric or adult oncology services [1,10]. Fortunately, awareness regarding this unique patient population has increased over time, leading to more AYA-dedicated care and research initiatives. In 2022, Husson et al. highlighted the importance of using patient-centered outcomes and the development of a core outcome set (COS), which is defined as “an agreed standardized collection of outcomes that should be measured and reported as a minimum”, for AYAs with cancer [11,12]. Several COSs are available for specific tumor types (such as breast, colorectal, prostate, and ovarian cancer), but these do not specifically capture the unique needs of AYAs with cancer as they do not specifically focus on age, but are developed for adults in general [12,13]. Therefore, there is a need for the development of an AYA-specific COS to supplement tumor-specific COSs. This will improve the relevance and efficiency of measuring outcomes, the pooling of (international) research data, and the value of care.

To address this, the European Union and Innovate UK funded the STRONG-AYA initiative, with the objective to develop and implement a COS for AYAs with cancer and disseminate the patient-centered outcomes to relevant stakeholders [14]. The development of a COS is based on the guidelines of COMET (Core Outcome Measures in Effectiveness Trials) and ICHOM (International Consortium for Health Outcomes Measurement) [15,16]. The first two steps in development involve a literature review and stakeholder interviews, and the results thereof form the input for a Delphi study. The full study protocol was described in the paper of Husson et al. (2024) [17].

The present literature review represents the first step in the development of a COS for AYAs and provides a comprehensive overview of all outcomes of potential relevance for AYAs with cancer represented in the international literature, including studies with a specific focus on AYAs and studies with AYA-stratified results.

## 2. Materials and Methods

For this literature review, the results were reported in line with the Preferred Reporting Items for Systematic Reviews and Meta-Analyses (PRISMA) guidelines where possible [18]. The review protocol is registered with OSF Registries (https://osf.io/vg2a3, accessed on 1 August 2023), the COS development process is registered in the COMET database [19], and the full study protocol is published online [17]. The latter describes the STRONG-AYA project in more detail.

### 2.1. Search Strategy

First, PROSPERO was checked to confirm that there are no similar reviews [17]. Once confirmed, a search string was designed by two team members (O.H. and S.H.M.J.) together with an experienced information specialist from the Erasmus University Medical Center (M.F.M.E.). The search string combined terms relating to (1) oncology and (2) adolescents and young adults. Appendix A includes the applied search strings per database. A literature search of the following databases was performed in October 2022 to identify relevant articles: Embase, MEDLINE ALL, Web of Science Core Collection, Cochrane Central Register of Controlled Trials, and additional search engines, including Google Scholar. The search was developed in Embase.com, optimized for sensitivity, and then translated to other databases following the method described by Bramer et al. [20]. The search strategies for Medline and Embase used relevant major thesaurus terms from Medical Subject Headings (MeSH) and Emtree, respectively. In all databases, terms were also searched in titles. Search terms were combined with Boolean operators AND and OR and proximity operators were used to combine terms into phrases. The searches in Embase, Medline, and Web of Science were limited to exclude conference papers. In all databases, animal-only articles and case reports were excluded from the search results. No study registries were searched, but Cochrane CENTRAL retrieves the contents of ClinicalTrials.gov and World Health Organization’s International Clinical Trials Registry Platform. The references were imported into EndNote and duplicates were removed by the information specialist using the method described by Bramer et al. [21].

### 2.2. Study Selection

Articles were eligible if they focused on adolescents and/or young adults with cancer (13–40 years old at initial cancer diagnosis) or included this age range among others and had age-stratified data available, regardless of the type of outcome. We allowed for both quantitative and qualitative studies to be included. There were no date restrictions. We excluded conference abstracts, reviews, editorials, case reports, publications of which the full text could not be obtained, and publications in any language other than English. Appendix B covers all applied in- and exclusion criteria. Rayyan, an online tool, was used to support the screening of publications and to record the reviewers’ screening decisions [22]. Due to the magnitude of this review, a team of reviewers was assembled, representing different positions (Masters students, PhD students, Postdoc researchers, and senior researchers) and areas of expertise (N = 14 for title/abstract screening and full-text screening, N = 15 for data extraction). Each review phase was preceded by an information and calibration session, covering the review guide (including project description and aims, contact information of the review coordinator, review process description, in- and exclusion criteria, definition overview, and planning), and a pilot exercise was performed to ensure consistency among reviewers. Weekly drop-in meetings were also scheduled for reviewers to discuss any ambiguities or difficulties. To reduce bias, during both title/abstract and full-text screening, two reviewers were paired and independently and blindly screened each publication against the eligibility criteria in Rayyan. In case of discrepancies after unblinding, these were resolved by the paired reviewers themselves together with/or by a third reviewer (S.H.M.J. and A.H.W.) if needed. Each phase was concluded with a feedback meeting to discuss successes and improvements.

### 2.3. Data Extraction

In order to standardize the data extraction among reviewers, a data extraction form was created based on expert input and previously used data extraction forms. It was pilot-tested beforehand on several articles accepted for data extraction. The following data were extracted verbatim per article (indicated by title, authors, and year of publication): type of study, study population at diagnosis, study population at study participation, phase of data collection, who reported the data (patient vs. proxy measure), country of study, sample size, tumor type(s), case-mix factor(s), outcome(s), and measurement tool(s). Case-mix factors refer to characteristics that can differ between healthcare systems and/or that can be predictive of outcomes, as described in the protocol paper [17]. For the outcomes, the revised taxonomy of Williamson and Clarke was used as input for the framework to categorize the exact outcomes in pre-specified core areas and outcome domains [23]. This ensured that similar outcomes with different terminology were grouped together, such as work, employment, and vocation. The framework covered 39 outcome domains, which were grouped in the following five core areas: epidemiology, physiological/clinical, functioning, resource use, and adverse events. For the case-mix factors, a predefined selection of case-mix categories was included in the extraction framework based on expert input. These included sex, age, partner status, educational level, social economic status (SES), migration background/ethnicity, children, tumor type, stage, metastases, treatment, and comorbidities (pre-existing and not effects of treatment). Each case-mix category had to occur at least four times in the dataset to be included as a separate category. If it occurred fewer than four times, the case-mix factor was categorized as ‘miscellaneous’. If it occurred four times or more, a new case-mix factor category was added (deductive approach). Tumor types were categorized based on an adapted taxonomy of van der Meer et al. [24], with clinical input from a medical doctor sought for clarification when necessary. Response options mainly included tick boxes and, if needed, open-text responses. The review coordinator (S.H.M.J.) was contacted in case of full-text requests or questions during all review phases. Measurement tools and case-mix factors were extracted as preliminary work for phase six (determine “how” to measure the COS) and phase seven (determine “case-mix” factors) of the COS development, respectively.

### 2.4. Quality Assurance

To assure the quality of the data extraction, an independent reviewer, who had not been part of the review before, was asked to extract data of a selection of the variables of at least one article of each reviewer who took part in the data extraction phase (representing the entire review team in the quality assurance). Articles were randomly selected using Random.org. The extracted data per article of both reviewers were compared to identify which variables were fairly straightforward to extract (such as tick boxes) vs. those that could potentially lead more easily to deviations (such as open-text boxes). Based on this, the review coordinator (S.H.M.J.) checked and cleaned the extracted data. If necessary, the principal investigator (O.H.) was consulted to discuss and solve any queries. After cleaning the full data extraction sheet, the data were analyzed.

## 3. Results

**Figure 1:**
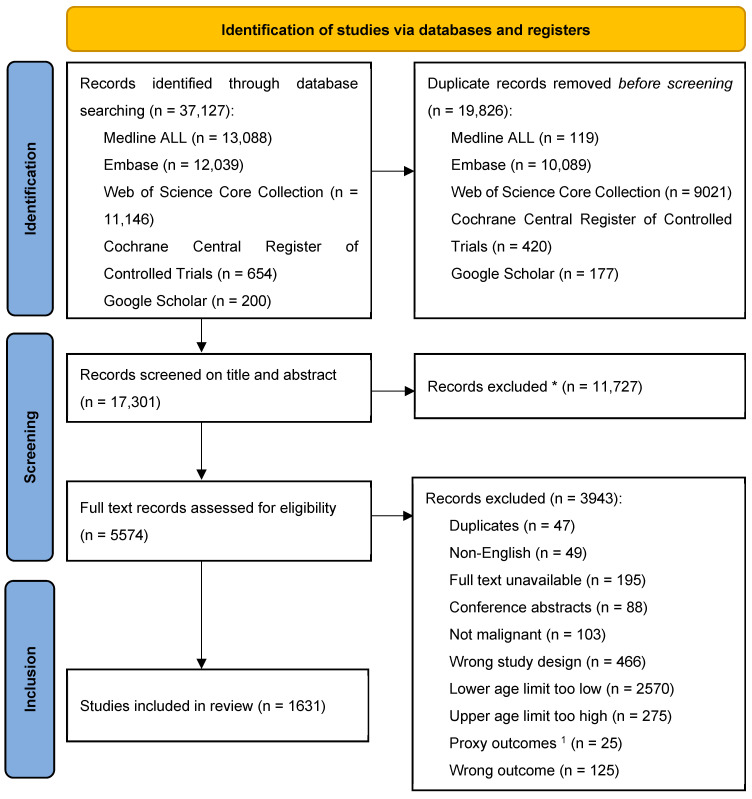
Flow chart of the article selection process. * No automation tools were used. ^1^ Articles describing outcomes of proxies solely and not those of AYAs were excluded.

Figure 1 illustrates the flow chart of the article selection process [25]. The literature search yielded 37,127 articles, which resulted in 17,301 unique articles after deduplication. These records were screened on title and abstract, leading to 5574 articles that met the criteria for full-text screening. After the full-text screening, 3943 articles were excluded, leading to the data extraction of 1631 articles.

### 3.1. Study Characteristics

**Figure 2:**
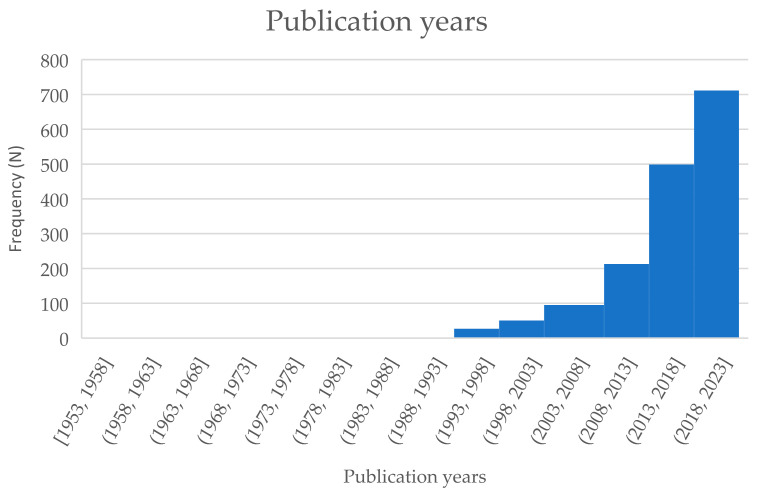
Publication years (per 5 years).

**Figure 3:**
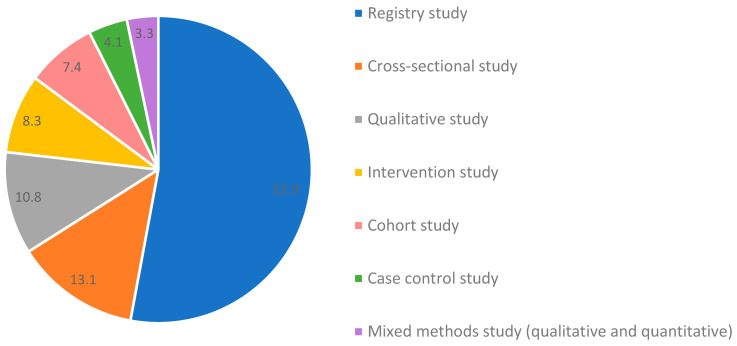
Distribution of study designs.

The 1631 included articles were published between 1953 and 2023 (Figure 2). Most articles have been published in the last 10 years. Of all articles, most were registry studies (52.9%), followed by cross-sectional (13.1%) and qualitative studies (10.8%) (Figure 3). Case–control (4.1%) and mixed-methods (3.3%) study designs were least frequently applied. The lower age range for age at diagnosis was, on average, 16 years [range: 13–36], and the upper age range was 32 years [range: 14–40]. More than two-thirds of the articles included proxy measures, which represented input from healthcare providers, relatives, and friends, as well as registry staff amongst others. AYAs were most often on treatment (73.4%) or within the first 5 years after diagnosis/treatment (49.9%) at the time of study participation (excluding registry studies). AYAs participated in research least often when they were in their palliative/end-of-life phase (3.3%). North America (50.3%) and Europe (27.8%) contributed the most in terms of number of articles, followed by Asia (17.2%) and Australia/New Zealand (4.6%), while South America (2.0%) and Africa (1.0%) provided the fewest articles. Most articles (54.9%) focused on one to five tumor types per article, in which leukemia (27.5%) and lymphomas (Hodgkin: 16.6% and non-Hodgkin: 13.6%) were most frequently represented. If more tumor types were represented, than there were rarely any restrictions to the tumor type selection.

### 3.2. Outcomes, Measurement Tools, and Case-Mix Factors

#### 3.2.1. Outcomes

Table 1 provides an overview of the most prevalent outcomes studied among AYAs with cancer and represented in the literature, stratified by core area and outcome domain. Of the five core areas, most outcomes were categorized within functioning (47.0%), epidemiology (44.9%), and physiological/clinical (42.4%), followed by resource use (6.1%) and adverse events (4.5%). Out of 39 outcome domains, the outcomes that were represented most frequently (>10.0%, independent of core area) included *mortality/survival* (43.1%), *delivery of care* (23.4%), *outcomes relating to neoplasms* (22.7%), and *emotional functioning/well-being* (19.2%). Given the variety of the level of detail and the applied terminology of the extracted outcomes, similar outcomes were merged into outcome categories to create a comprehensive outcome overview. The last column of Table 1 includes these outcomes, which are in order of prevalence (descending); per outcome domain, the top three most prevalent outcomes were established, followed by the less prevalent outcomes. Supplementary File S1 provides a full data extraction overview, including the exact outcomes.

#### 3.2.2. Measurement Tools

The extracted measurement tools were very diverse, but trends were seen based on study design and core area (Supplementary File S1). As expected, articles with outcomes categorized in the epidemiology core area, derived mostly from registry studies, used medical records and local and/or national (cancer-specific) registries more often, and, to a lesser extent, clinical trial data. A commonly used registry is the Surveillance, Epidemiology, and End Results (SEER) registry. Conversely, outcomes of the functioning core area were more often seen in studies using patient-reported information and, to a lesser extent, registry data and qualitative data (i.e., interviews, focus groups). Many of the patient-reported outcome measures (PROMs) represented in this review were frequently used, validated questionnaires such as the Hospital Anxiety and Depression Scale (HADS), the Pediatric Quality of Life Inventory (PedsQL), the European Organization for Research and Treatment of Cancer Quality of Life Group C30 (EORTC QLQ-C30), the Brief Symptom Inventory (BSI), and the Short Form Survey (SF-12 or SF-36). However, some questionnaires were developed for a particular study. The remaining core areas (physiological/clinical, resource use, and adverse events) cover a mixture of measurement tools, although data collected by registries seemed to be used slightly more often in studies concerning these areas.

#### 3.2.3. Case-Mix Factor(s)

Table 2 provides an overview of the case-mix factors represented in the articles. An inductive approach was applied to decide which categories needed to be created. Input from medical doctors was received to categorize clinical data. Each case mix is a collective factor, representing terms of similar topics.

## 4. Discussion

This literature review provides a comprehensive overview of all the relevant outcomes for AYAs with cancer represented in the international literature and is the first step in the development of an AYA-specific COS [12,17]. Despite the enormous diversity among the extracted variables, our results show several trends. Most articles were labeled as registry studies and used proxy measures (e.g., registry data, medical records). The majority of the data came from North America or Europe. The prevailing outcome domains included *mortality/survival, delivery of care, outcomes relating to neoplasms,* and *emotional functioning/well-being*. The results of this review, together with stakeholder interviews, serve as input for a Delphi study to develop a COS for AYAs with cancer. However, it is important to note that the COS is not static; therefore, further development and refinement may occur once created.

It should be noted that the outcomes represented in this review may not all be AYA-specific per se because some outcomes may be more cancer-generic or tumor-specific. Ideally, a cancer-generic COS can be used alongside tumor-specific and/or age-specific COSs. For example, tumor-specific COSs for breast, colorectal, prostate, and ovarian cancer have been developed [13]. However, as AYA cancer patients may face (partly) similar issues as, but also distinct issues from, older patients, the addition of an age-specific COS can help to identify the unique issues and needs of both younger and older cancer patients more effectively [26]. Unfortunately, there is no cancer-generic all-ages COS at present, and only limited tumor-specific COSs are available. This scarcity and further refinement of the AYA-specific COS should be evaluated over time. As a future exercise, it may be insightful to define which outcomes are age-specific, cancer-generic, and/or tumor-specific based on the input of different stakeholders (i.e., AYAs/relatives, healthcare providers, researchers). However, for now, it is important that the entire spectrum of relevant outcomes is represented in the AYA-specific COS, as there are no cancer-generic and more tumor-specific COSs currently available.

The measurement tools represented in the review were heterogeneous, including objective tools such as registry data and medical records, as well as PROMs and qualitative measures. For some outcomes, the choice of the most appropriate tool is a straightforward decision (such as an objective measure for blood pressure or PRO measure for quality of life), while, for others, more possibilities exist. Many studies used validated, but also cancer-generic tools. However, these measures are not specific enough to determine the relevant outcomes or to capture differences between groups or changes over time. Having age-specific measurement tools or domains available can prevent these both from happening [5,27]. For example, employment can be of importance to both younger and older patients but, for AYAs, career establishment can be of greater concern, while, for older patients, early retirement may be more relevant. Fortunately, several organizations are currently working on these age-specific initiatives, like the PROMIS AYA and EORTC QLG AYA initiatives [5,27]. Ideally, there should be no restrictions as to which validated tool to use to measure a specific outcome; rather, there should be several options available, enabling a comparison of outcome results [13,28]. With this, it is also important to take into account the burdening of patients, including repetitive or irrelevant questions. Regardless of the choice of tool, one would be able to compare results over time, between groups or countries, for example. However, to enable these comparisons, measurement tools of similar outcomes need to be calibrated [29]. Future research should perform calibration exercises to enable the global usage and comparison of different tools. This should be considered as an important next step in research.

The revised framework of Williamson and Clarke, which we used in a slightly adapted form to categorize our outcomes, has been used previously in the development of other COS initiatives [13,23]. The latter was the reason to use this framework, in order to potentially align our AYA-specific COS with other (future) COSs. Additionally, we can use the extracted data to perform offshoot reviews to focus on data subsets, such as specific outcomes and tumor types. However, the framework of Williamson and Clarke is not age-specific, which made the categorization of outcomes somewhat difficult. Some outcome domains were rarely or not represented at all, such as *injury and poisoning outcomes* and *societal/carer burden* (due to the focus on AYAs), while other domains represent a rather broad range of outcomes, such as *physical functioning*. Additionally, some outcomes were closely related or potentially overlapped; however, these were categorized in different domains or could also have been categorized across several domains. For example, health behaviors such as exercising, sun exposure, eating behavior, and drug use were classified within the *physical functioning* domain. However, one could argue that these concepts should be categorized into a different category or that they align with other domains, such as *social functioning*. Similarly, there were three circulatory system outcome domains (*blood and lymphatic system outcomes* (1.8%), *cardiac outcomes* (1.0%), and *vascular outcomes* (0.8%)) that all refer to closely related outcomes, but were categorized as three different domains. Another example is diabetes mellitus, which was categorized within the outcome domain *metabolism and nutrition outcomes*, but it could also be categorized in the domain *endocrine outcomes*. Regardless of the outcome domain in which an outcome was categorized, it is of most importance that it is represented in the COS if needed.

### 4.1. Future Perspectives

Once the COS is developed, the next aim of the STRONG-AYA project is to implement the COS in several countries, such as the Netherlands, the United Kingdom, France, Italy, and Poland to begin with [12,17]. Collecting and analyzing both retrospective and prospective data will provide new insights in order to improve the outcomes and care for AYAs with cancer. Measuring similar outcomes over time in different countries will enable us to look for opportunities to improve outcomes at several levels, including the patient, cancer center, and (inter)national level (dissemination). The multi-stakeholder involvement and approach of STRONG-AYA, representing AYAs with cancer, healthcare providers, and researchers, among others, will contribute to a more efficient and effective dissemination.

As previously discussed, it is important to keep the COS up to date. This refers both to the possible alignment and use of an AYA-specific COS with other COSs, as well as the content of the COS. For this, additional research is needed. The AYA-specific COS should be further refined, for example, based on stages of the patient journey (patient on treatment versus survivor off treatment), multimodality treatments, tumor types (age-specific epidemiology), representation of minority groups (based on health illiteracy or countries, for example), or country/treatment centers. As an example, most articles were based on data from North America and Europe (i.e., high-income countries), leading to the under-representation of many other countries (i.e., low- and middle-income countries)/cultures and, with that, outcomes relevant to them. If the COS is going to be implemented in more countries (also outside of Europe), both the content as well as the format should be re-evaluated and adapted.

### 4.2. Strengths and Limitations

A major strength of this extensive and comprehensive review is the team of reviewers, which enabled a rigorous approach and the screening of more than 17,000 articles included in this literature review. During the different review phases, the team was guided by tailored review documentation, calibration and information sessions, and Q&A drop-in meetings. Other strengths include the use of Rayyan (an online screening tool that allows for blinding and pairing reviewers), the quality assurance, and the development and use of a pilot-tested, customized extraction sheet (partly based on predefined classifications for outcomes and tumor types). Lastly, the search strategy was developed together with an experienced information specialist.

However, this literature review is not without limitations. Firstly, we may have missed relevant outcomes due to the (strictness of the) applied age range at initial cancer diagnosis (13–40 years old at initial cancer diagnosis). The (lack of) information in some articles, such as missing age at diagnosis, may have led to the exclusion of articles that could have been relevant. With the widened, applied age range at initial cancer diagnosis, as agreed on by our consortium of AYA experts, we aimed to be as inclusive as possible and prevent the exclusion of relevant articles. Secondly, some case-mix factors and outcomes are closely related to each other or act as a collective factor. For example, the case-mix factor *treatment* covers the following amongst others: having received treatment or not, being on or off treatment, the type of treatment received, the dose received, and the timing of the treatment. In line with this, we chose to categorize each study (article) to a maximum of one study design, which might have led to an under-representation of some study designs. Thirdly, although the team of reviewers was a strength, the reviewers were also diverse in expertise and experience with reviewing. We aimed to align the reviewers on screening and data extraction by developing a review guide, organizing calibration and information sessions and Q&A drop-in meetings, and blinded, double reviewing. However, even with these initiatives and the quality assurance and cleaning phase, we might not have been able to prevent differences in screening and extraction between reviewers. This may have impacted the selected, extracted, and analyzed data. Fourthly, due to the magnitude of the project, the timelines were stretched. In line with the general recommendation for COS development and updating the (input for) the COS, it is advised to perform an updated search and review at a later point in time. This is also of importance as outcomes may change based on future changes in treatment. Lastly, we only evaluated the literature in English and may have missed relevant outcomes for non-English-speaking areas of the world.

## 5. Conclusions

This comprehensive literature review is unique as it is the world’s largest AYA-dedicated literature review, bringing together PROs and objective/clinical outcomes. It provides a foundation for the development of an AYA-specific COS. This will help to improve the relevance and efficiency of measuring outcomes, the pooling of (international) research data, and the value of care for current and future AYAs with cancer. Eventually, the finalized COS will be implemented in healthcare and research systems at an international level. Further refinement of the COS should be a priority for future research initiatives.

## Figures and Tables

**Table 1:** Overview of outcomes of relevance among AYAs with cancer represented in the international literature, based on the revised framework of Williamson and Clarke.

Core Area	% *	Outcome Domain	% *	Outcome Categories (Descending Prevalence)
Epidemiology	44.9	Mortality/survival	43.1	Overall survival
				Survival (specified and non-specified)
				Event-free survival
				Other: Mortality (specified and non-specified); Disease-free survival; Progression-free survival; Cancer-specific survival; Relative survival; Relapse-free survival; Recurrence-free survival; Cancer-specific mortality; Disease-specific death; Disease-specific survival; Metastasis-free survival; Standard mortality ratio; All-cause mortality; Treatment-related mortality; Excess mortality/conditional survival; Death hazard ratio; Overall mortality; Miscellaneous
		Incidence ^1^	8.2	Cancer incidence
				Prevalence
				Miscellaneous
Physiological/clinical	42.4	Blood and lymphatic system outcomes	1.8	Blood markers
				Hematologic diseases (specified and non-specified)
				Thrombosis
				Other: Lymphatic outcomes; Miscellaneous
		Cardiac outcomes	1.0	Cardiac diseases (specified and non-specified)
				Incidence of cardiac diseases
				Miscellaneous
		Congenital, familial, and genetic outcomes	5.3	Gene expression/mutations
				Family history
				Genetic testing
				Other: Miscellaneous
		Endocrine outcomes	1.5	Endocrine diseases (specified and non-specified)
				Thyroid diseases
				Menopausal outcomes
				Other: Miscellaneous
		Ear and labyrinth outcomes	0.2	Hearing loss
				Ototoxicity
		Eye outcomes	0.2	Diseases relating to vision (specified and non-specified)
				Posterior scleral bowing
				Cataracts
		Gastrointestinal outcomes	0.9	Appetite
				Constipation
				Mouth issues
				Other: Vomiting; Diarrhea; Swallowing; Dental problems; Gastrointestinal diseases (specified and non-specified); Miscellaneous
		General outcomes	6.4	Fatigue
				Symptoms (specified and non-specified)
				Pain
				Other: Sleep; Nausea; Anthropometric outcomes; General health; Dyspnea; Dizziness; Miscellaneous
		Hepatobiliary outcomes	0.4	Cirrhosis
				Chronic liver diseases
				Liver transplantation
				Other: Liver diseases (specified and non-specified); Miscellaneous
		Immune system outcomes	1.2	Graft versus host disease (GvHD) outcomes
				Immune system diseases (specified and non-specified)
				Human immunodeficiency virus (HIV)
				Other: Miscellaneous
		Infection and infestation outcomes	1.2	Infections (specified and non-specified)
				Pancreatitis
				Helicobacter pylori infections (HPIs)
				Other: Epstein–Barr virus (EBV); Human papilloma virus (HPV); Miscellaneous
		Injury and poisoning outcomes	0.0	
		Metabolism and nutrition outcomes	0.9	Diabetes mellitus
				Metabolic outcomes (specified and non-specified)
				Dietary outcomes
				Other: Miscellaneous
		Musculoskeletal and connective tissue outcomes	0.7	Musculoskeletal outcomes (specified and non-specified)
				Osteonecrosis
				Strength and flexibility outcomes
				Other: Miscellaneous
		Outcomes relating to neoplasms: benign, malignant, and unspecified (including cysts and polyps)	22.7	Stage
				Tumor characteristics (specified and non-specified)
				Treatment characteristics (specified and non-specified)
				Other: Tumor type; Metastases; Disease status; Relapses; Recurrences; Location; Second malignancies; Time intervals; Symptoms (specified and non-specified); Subsequent malignant neoplasms; Miscellaneous
		Nervous system outcomes	0.9	Nervous system outcomes (specified and non-specified)
				Stroke
				Seizure
				Other: Neurotoxicity; Miscellaneous
		Pregnancy, puerperium, and perinatal outcomes	2.0	Childbirth outcomes
				Pregnancy outcomes (specified and non-specified)
				Baby/offspring characteristics (specified and non-specified)
				Other: Characteristics of the delivery (specified and non-specified); Maternal outcomes; Desire for children; Miscellaneous
		Renal and urinary outcomes	0.9	Renal diseases (specified and non-specified)
				Treatment characteristics
				Urinary outcomes (specified and non-specified)
				Other: Miscellaneous
		Reproductive system and breast outcomes	6.7	Fertility (specified and non-specified)
				Preservation
				Emotions
				Other: Information provision/counseling; Sexual health; Knowledge; Menstrual outcomes; Contraceptive use; Breast outcomes; Miscellaneous
		Psychiatric outcomes	0.6	Suicide
				Psychiatric outcomes (specified and non-specified)
				Miscellaneous
		Respiratory, thoracic, and mediastinal outcomes	1.2	Respiratory diseases (specified and non-specified)
				Shortness of breath
				Chronic obstructive pulmonary disease (COPD)
				Other: Asthma; Pulmonary fibrosis; Miscellaneous
		Skin and subcutaneous tissue outcomes	0.1	Skin changes
		Vascular outcomes	0.8	Vascular outcomes (specified and non-specified)
				Blood pressure
				Miscellaneous
Functioning	47.0	Physical functioning	9.7	Physical health
				Physical functioning
				Physical activity
				Other: Health behavior (specified and non-specified); Smoking; Sexual functioning; Alcohol; Drugs; Dietary behavior; Performance status; Appearance; Miscellaneous
		Social functioning	9.7	Social functioning
				Social support
				Psychosocial health
				Other: Online support; Social involvement in care-related decisions; Social needs; Isolation; Disclosure; Miscellaneous
		Role functioning	6.3	Employment
				School
				Role functioning
				Other: Partner outcomes; Functional well-being; Intimacy; Miscellaneous
		Emotional functioning/well-being	19.2	Distress
				Mental health
				Depression
				Other: Anxiety; Emotional functioning; Concerns; Coping; Worry; Body image; Fear of cancer recurrence; Self-efficacy; (Post-traumatic) Growth; Post-traumatic stress; Hope; Needs; Stress; Mental support; Fear; Self-esteem; Satisfaction; Cancer-related meaning; Uncertainty; Loneliness; Identity; Resilience; Regret; Psychosocial experiences; Identity; Body image; Miscellaneous
		Cognitive functioning	5.8	Cognitive functioning
				Spiritual
				Knowledge
				Other: Religious outcomes; Beliefs; Attitudes; Meaning of life; Cognitive health; Literacy; Goal setting; Concentration; Memory; Miscellaneous
		Global quality of life	5.6	Quality of life
		Perceived health status	3.1	Cancer experience
				Impact of cancer
				Health status
				Other: Future; Participation in life; Miscellaneous
		Delivery of care	23.4	Healthcare use
				Needs
				Fertility-related care
				Other: Trial participation/availability; Care experiences; Care preferences; Time intervals; Acceptability; Characteristics of care center; Perceptions; Adherence; Palliative care; Communication outcomes; Satisfaction with care; Pediatric and/or/vs. adult care; Source of information; Attendance to care; Documentation of care; Compliance; Miscellaneous
		Personal circumstances	4.5	Financial burden
				Insurance
				Expenditures
				Other: Financial distress; Benefits; Practical challenges; Living situation; Miscellaneous
Resource use	6.1	Economic	0.8	Costs
				Cost-driven behavior
				Miscellaneous
		Hospital	2.3	Hospitalizations
				Length of stay
				Emergency department care
				Other: Finances; Miscellaneous
		Need for further intervention	3.1	Feasibility
				Efficacy
				Validation
				Other: Safety; Accuracy; Evaluation; Reliability; Miscellaneous
		Societal/carer burden	0.0	
Adverse events	4.5	Adverse events	4.5	Toxicity
				Complications (specified and non-specified)
				Adverse events (specified and non-specified)
				Other: Morbidity; Miscellaneous

* Percentage of articles in the review that include outcomes that fall into the corresponding core area (column 2) or outcome domain (column 4). The reference group is the total number of included articles (N = 1631). ^1^ Incidence was only included when it was not the sole outcome.

**Table 2:** Case-mix factors.

Nr.	Case-Mix Factors *(Examples)*	N	% *
**1**	**Age** *(age at diagnosis; age at study participation)*	897	74.7
**2**	**Sex** *(sex; gender)*	711	59.2
**3**	**Malignant (sub)types** *(tumor types; histological subtypes; date of diagnosis)*	667	55.5
**4**	**Treatment** *(type of treatment; number of treatments; treatment dosage; date of treatment)*	488	40.6
**5**	**Stage** *(stage; TNM classification; tumor size; metastasis; prognosis)*	473	39.4
**6**	**Ethnicity** *(race; migration background; ethnicity)*	314	26.1
**7**	**Socioeconomic status (SES)** *(socioeconomic position; median income)*	190	15.8
**8**	**Education** *(educational level; current or highest grade in school; change in school life)*	146	12.2
**9**	**Partner** *(partner status; marital status; sexual orientation)*	140	11.7
**10**	**Delivery of care** *(type of treatment center; location of center; patient provider communication; healthcare access; unmet service needs)*	87	7.2
**11**	**Insurance** *(insurance status; insured by Medicaid; insurance provider)*	85	7.1
**12**	**Comorbidities (pre-existing and not effects of treatment)** *(comorbidities; pre-treatment mental health diagnosis)*	80	6.7
**13**	**Geographical area/residence** *(urban/rural residence; (place of) residence; distance to care center)*	81	6.7
**14**	**Symptoms** *(symptoms, complaints; duration of symptoms; complications; late effects)*	74	6.2
**15**	**Genetics** *(family history of cancer; cytogenetic/molecular genetic characteristics; genetic mutations)*	66	5.5
**16**	**Tumor markers** *(white blood cell count; serum levels; expression of tumor markers)*	61	5.1
**17**	**Children** *(having children; caregiving for children at diagnosis)*	56	4.7
**18**	**Location (of tumor)** *(primary tumor site; tumor location)*	54	4.5
**19**	**Employment** *(being employed, type of work; change in work life)*	52	4.3
**20**	**Lifestyle** *(physical activity; sleep; alcohol intake; smoking status)*	50	4.2
**21**	**Health status** *(health status; performance status; functional status)*	39	3.2
**22**	**Maternity/fertility characteristics** *(ever been pregnant; maternal age; infertility; menarche status)*	35	2.9
**23**	**Anthropometric measures** *(obesity status; body mass index)*	32	2.7
**24**	**Psychological mechanisms** *(anxiety, depression; psychological outcomes; coping)*	33	2.7
**25**	**Secondary malignancies** *(second malignancies; history of cancer; stage of second malignancy)*	30	2.5
**26**	**Sociodemographic characteristics** *(living status; language; religion; personality; health literacy; family structure)*	26	2.2
**27**	**Recurrence/relapse** *(recurrence; relapse; site of recurrence; time since relapse)*	25	2.1
**28**	**Social support** *(social support; social factors; social desirability)*	17	1.4
**29**	**Registry characteristics** *(type of registry or cohort; survey year)*	11	0.9
**30**	**Virus/bacteria** *(HIV status; HPV status)*	10	0.8
**31**	**Trial participation** *(clinical/study trial participation; clinical trial enrollment)*	6	0.5
**32**	**COVID-19 characteristics** *(pre/post COVID-19 pandemic; adherence to COVID-19 precautions; COVID impact)*	4	0.3
**33**	**Miscellaneous** *(communication; stressful life event(s))*	25	2.1

* Of total number of articles with case-mix factor(s) (N = 1201).

## Data Availability

The data presented in this study are available upon reasonable request from the corresponding author.

## Supplementary Materials

The following supporting information can be downloaded at: https://www.mdpi.com/article/10.3390/cancers17030454/s1, Supplementary File S1: Data Extraction Overview.

## Abbreviations

The following abbreviations are used in this manuscript:

AYA	Adolescent and young adult
COS	Core outcome set
COMET	Core Outcome Measures in Effectiveness Trials
ICHOM	International Consortium for Health Outcomes Measurement
PRISMA	Preferred Reporting Items for Systematic Reviews and Meta-Analyses
MeSH	Medical Subject Headings
Nr	Number
SES	Social economic status
GvHD	Graft versus host disease
HIV	Human immunodeficiency virus
HPIs	Helicobacter pylori infections
EBV	Epstein–Barr virus
HPV	Human papilloma virus
COPD	Chronic obstructive pulmonary disease
SEER	Surveillance, Epidemiology, and End Results
PROMs	Patient-reported outcome measures
HADS	Hospital Anxiety and Depression Scale
PedsQL	Pediatric Quality of Life Inventory
EORTC QLQ-C30	European Organization for Research and Treatment of Cancer Quality of Life Group C30
BSI	Brief Symptom Inventory
SF	Short Form Survey
COVID-19	Corona virus disease

## Appendix A. Search String for the Literature Review per Database

Embase: (‘malignant neoplasm’/mj/exp OR ‘neoplasm’/mj/de OR ‘oncology’/mj/exp OR ‘metastasis’/mj/exp OR ‘myelodysplastic syndrome’/mj/de OR ‘cancer patient’/mj/exp OR ‘digestive system tumor’/mj/exp OR ‘cancer mortality’/mj/de OR ‘cancer survival’/mj/exp OR ‘cancer diagnosis’/mj/exp OR (neoplas* OR cancer* OR malign* OR tumor* OR tumour* OR carcinoma* OR oncolog* OR melanom* OR sarcoma* OR leukemi* OR leukaemi* OR lymphoma* OR glioblastoma* OR metastas* OR myelom* OR hepatoblastoma* OR medulloblastoma* OR glioma* OR myelodysplastic* OR liposarcom* OR osteosarcoma* OR rhabdomyosarcoma* OR adenocarcinoma* OR chordoma* OR aml OR nhl OR craniopharyngioma* OR Hodgkin-disease* OR hepatoma* OR myeloma* OR multiple-myeloma* OR blastoma* OR neuroblastoma* OR ganglioneuroblastoma* OR chondrosarcoma*):ti) AND (‘young adult’/mj/de OR ‘adolescent’/mj/de OR (juvenil* OR adolescen* OR preadolescen* OR youth* OR young-adult* OR teen* OR puber* OR pubescen* OR highschool* OR AYA OR TYA OR young*-patient*):ti) NOT ([Conference Abstract]/lim OR [Conference Review]/lim) NOT ([animals]/lim NOT [humans]/lim) NOT (‘case report’/de OR ‘editorial’/de OR (case-report):ti) AND [ENGLISH]/lim

Medline: (exp *Neoplasms/OR exp *Medical Oncology/OR exp *Myelodysplastic Syndromes/OR *Cancer Survivors/OR *Early Detection of Cancer/OR (neoplas* OR cancer* OR malign* OR tumor* OR tumour* OR carcinoma* OR oncolog* OR melanom* OR sarcoma* OR leukemi* OR leukaemi* OR lymphoma* OR glioblastoma* OR metastas* OR myelom* OR hepatoblastoma* OR medulloblastoma* OR glioma* OR myelodysplastic* OR liposarcom* OR osteosarcoma* OR rhabdomyosarcoma* OR adenocarcinoma* OR chordoma* OR aml OR nhl OR craniopharyngioma* OR Hodgkin-disease* OR hepatoma* OR myeloma* OR multiple-myeloma* OR blastoma* OR neuroblastoma* OR ganglioneuroblastoma* OR chondrosarcoma*).ti.) AND (*Young Adult/OR *Adolescent/OR (juvenil* OR adolescen* OR preadolescen* OR youth* OR young-adult* OR teen* OR puber* OR pubescen* OR highschool* OR AYA OR TYA OR young*-patient*).ti.) NOT (news OR congres* OR abstract* OR book* OR chapter* OR dissertation abstract* OR editorial*).pt. NOT (exp animals/NOT humans/) NOT (Case Reports/OR (case-report).ti.) AND english.la.

Cochrane: ((neoplas* OR cancer* OR malign* OR tumor* OR tumour* OR carcinoma* OR oncolog* OR melanom* OR sarcoma* OR leukemi* OR leukaemi* OR lymphoma* OR glioblastoma* OR metastas* OR myelom* OR hepatoblastoma* OR medulloblastoma* OR glioma* OR myelodysplastic* OR liposarcom* OR osteosarcoma* OR rhabdomyosarcoma* OR adenocarcinoma* OR chordoma* OR aml OR nhl OR craniopharyngioma* OR Hodgkin-disease* OR hepatoma* OR myeloma* OR multiple-myeloma* OR blastoma* OR neuroblastoma* OR ganglioneuroblastoma* OR chondrosarcoma*):ti) AND ((juvenil* OR adolescen* OR preadolescen* OR youth* OR young-adult* OR teen* OR puber* OR pubescen* OR highschool* OR AYA OR TYA OR young-patient*):ti) NOT “conference abstract”:pt

Web of Science: TI = (((neoplas* OR cancer* OR malign* OR tumor* OR tumour* OR carcinoma* OR oncolog* OR melanom* OR sarcoma* OR leukemi* OR leukaemi* OR lymphoma* OR glioblastoma* OR metastas* OR myelom* OR hepatoblastoma* OR medulloblastoma* OR glioma* OR myelodysplastic* OR liposarcom* OR osteosarcoma* OR rhabdomyosarcoma* OR adenocarcinoma* OR chordoma* OR aml OR nhl OR craniopharyngioma* OR Hodgkin-disease* OR hepatoma* OR myeloma* OR multiple-myeloma* OR blastoma* OR neuroblastoma* OR ganglioneuroblastoma* OR chondrosarcoma*)) AND ((juvenil* OR adolescen* OR preadolescen* OR youth* OR young-adult* OR teen* OR puber* OR pubescen* OR highschool* OR AYA OR TYA OR young*-patient*)) NOT ((animal* OR rat OR rats OR mouse OR mice OR murine OR dog OR dogs OR canine OR cat OR cats OR feline OR rabbit OR cow OR cows OR bovine OR rodent* OR sheep OR ovine OR pig OR swine OR porcine OR veterinar* OR chick* OR zebrafish* OR baboon* OR nonhuman* OR primate* OR cattle* OR goose OR geese OR duck OR macaque* OR avian* OR bird* OR fish*) NOT (human* OR patient* OR women OR woman OR men OR man))) NOT DT = (Meeting Abstract OR Meeting Summary) NOT TI = (case-report) NOT DT = (Editorial Material) AND LA = (English)

Google Scholar:

AYA|TYA|adolescents|adolescent|“young adult|adults” neoplasm|cancer|malignancy|tumor|tumour|carcinoma|oncology|melanoma|sarcoma|leukemia|leukaemia|lymphoma|glioblastoma|metastasis|myeloma

AYA|TYA|adolescents|adolescent|‘young adult|adults’ neoplasm|cancer|malignancy|tumor|tumour|carcinoma|oncology|melanoma|sarcoma|leukemia|leukaemia|lymphoma|glioblastoma|metastasis|myeloma

## Appendix B. In- and Exclusion Criteria

Inclusion criteria:

- Population: AYAs (13 up until (and including) 40 years old at initial cancer diagnosis) or a subset of this age range (e.g., adolescents OR young adults only). The AYA age range will be flexibly applied, because lower and upper age limits for AYAs differ per country or per study;
- Mixed samples will be included if age-stratified outcomes are available for the target population (i.e., AYAs; adolescents; young adults);
- Studies conducted in other study populations, such as healthcare providers, friends, parents, or carers of AYAs, will be included only if the participants provide information on the outcomes of AYAs;
- Study population at study participation: on and/or off treatment, including at diagnosis, during treatment, or following treatment (patients and/or survivors). There is no limit for the time since diagnosis for AYA cancer survivors. Patients on maintenance treatment will be included;
- Written in English language;
- Any type of malignant tumor;
- Study designs: prospective intervention studies, randomized controlled trials (RCTs), observational cohort studies, case–control studies, cross-sectional studies, qualitative studies, registry studies, mixed-methods studies (qualitative and quantitative methods);
- Studies focusing on all types of biological, physical, psychological, or social outcomes.

Exclusion criteria:

- Non-English articles;
- Full text is unavailable;
- Conference abstracts or posters;
- Article focusing on non-malignant tumor type(s);
- Non-human study;
- Study population consisting exclusively of adolescent and young adult participants without a cancer diagnosis;
- Articles describing study protocols, case reports/series, reviews/meta-analyses, expert opinions, theoretical papers, policy documents/guidelines, consensus letters, editorials;
- Study population consisting exclusively of childhood cancer patients aged under 13 years at initial cancer diagnosis and/or adult cancer patients aged over 40 years at initial cancer diagnosis;
- Study population consisting of childhood and AYA cancer patients OR AYA and adult cancer patients with NO age-stratified outcomes available (i.e., AYA-specific outcomes cannot be identified);
- Articles focusing on outcomes not of interest (such as solely focusing on the outcome incidence).
